# Dysregulation of long non-coding RNA in breast cancer: an overview of mechanism and clinical implication

**DOI:** 10.18632/oncotarget.12537

**Published:** 2016-10-08

**Authors:** Ji Wang, Chenyang Ye, Hanchu Xiong, Yong Shen, Yi Lu, Jichun Zhou, Linbo Wang

**Affiliations:** ^1^ Department of Surgical Oncology, Sir Run Run Shaw Hospital, Zhejiang University, Hangzhou, Zhejiang, China; ^2^ Biomedical Research Center and Key Laboratory of Biotherapy of Zhejiang Province, Hangzhou, Zhejiang, China; ^3^ Cancer Institute (Key Laboratory of Cancer Prevention & Intervention, National Ministry of Education), Second Affiliated Hospital, Zhejiang University, Hangzhou, Zhejiang, China; ^4^ Zhejiang University School of Medicine, Hangzhou, Zhejiang, China

**Keywords:** breast cancer, long non-coding RNA, mechanism, biomarker, treatment target

## Abstract

Long non-coding RNAs (lncRNAs), which occupy nearly 98% of genome, have crucial roles in cancer development, including breast cancer. Breast cancer is a disease with high incidence. Despite of recent progress in understanding the molecular mechanisms and combined therapy strategies, the functions and mechanisms of lncRNAs in breast cancer remains unclear. This review presents the currently basic knowledge and research approaches of lncRNAs. We also highlight the latest advances of seven classic lncRNAs and three novel lncRNAs in breast cancer, elucidating their mechanisms and possible therapeutic targets. Additionally, association between lncRNA and specific molecular subtype of breast cancer is reported. Lastly, we briefly delineate the potential roles of lncRNAs in clinical applications as biomarkers and treatment targets.

## INTRODUCTION

Over the last few decades, accumulating evidence has suggested the importance of non-coding RNAs (ncRNAs) in the regulation of biological processes, such as development, differentiation, metabolism and metastasis [1]. The ncRNAs can be divided into two categories according to length: one category is small ncRNAs ( < 200 bp), including tRNA, rRNA, microRNA, snoRNA, and Piwi-interacting RNA, etc. [2–5]; and the other one is long non-coding RNAs (lncRNAs) ( > 200 bp) [6].

LncRNAs can be further classified according to its genetic location and transcriptional direction. Sense lncRNAs entirely or partially overlap with nearby coding gene, whereas intergenic lncRNAs do not overlap. Both of them are transcribed from the DNA template strand. In contrast, antisense lncRNAs and divergent lncRNAs originate from the non-template strand. Antisense lncRNAs overlap with mRNA gene body, and divergent lncRNAs share promoter with nearby coding genes but extend in an opposite orientation [7]. After being transcribed by RNA polymerase II, all of these lncRNAs are 5′-capped, 3′-polyadenylated, spliced and some of them will be exported to cytoplasm [7]. It is recently reported that lncRNAs may contain short open reading frame (sORF, less than 100 codons) and hence produce small peptides, although these peptides used to be neglected by classic open reading frame prediction based on a cutoff of 100 codons as minimum length [8–10].

LncRNAs exert function in either nucleus or cytoplasm, sometimes in both. Nuclear lncRNAs participate in chromatin remodeling and modification, transcriptional modulation, or RNA processing, while cytoplasmic lncRNAs usually interact with mature mRNA and/or protein [11, 12].

## MOLECULAR MECHANISMS OF LNCRNAS

Several classification systems have been built to better arrange the mechanisms of lncRNA activity. One of them sorts lncRNAs into four archetypes: 1) signal; 2) decoy; 3) guide; and 4) scaffold [13]. 1) The signal lncRNAs are transcribed when certain condition stimulates their DNA elements. These conditional transcripts, such as X-inactive-specific-transcript (XIST) and HOX antisense intergenic RNA (HOTAIR), usually serve as markers for biological events and are capable of delivering further signals. 2) The decoy archetype, such as growth arrest specific 5 (GAS5) and metastasis-associated lung adenocarcinoma transcript 1 (MALAT1), possesses an RNA motif which is similar to its DNA counterpart. They decoy DNA binding proteins, but exert no additional function. Some lncRNAs acting as a “sponge” and sinking miRNAs also belong to this type. 3) The guide lncRNAs guide functional molecules to specific DNA sequences, either in cis (affecting nearby genes) or in trans (affecting distant genes). XIST and HOTAIR are typical examples, respectively. 4) If one lncRNA owns multiple domains that combine several molecules together, it would be sorted into scaffold archetype, like HOTAIR [14]. As a whole, these archetypes are not mutually exclusive, and one single lncRNA may perform its function based on a combination of the above mechanisms. Another classification system is based on the levels on which lncRNAs have influenced, such as epigenetic modification, transcriptional modulation and post-transcriptional regulation.

## RNA-CENTRIC APPROACHES *IN VITRO*

Classical assays (such as knockdown, knockout, rescue, and overexpression, etc.) have contributed to explore the world of lncRNAs [13]. Recently, many novel RNA-centric approaches are emerging. These technologies mainly focus on studying location, interaction partner and structure of lncRNAs [15] (summarized in Table 1). For instance, chromatin isolation by RNA purification (CHIPR), capture hybridization analysis of RNA targets (CHART) and RNA antisense purification (RAP) are three new tools to map lncRNA-binding sites in the genome. One of them, CHIPR, uses biotinylated tiles of oligonucleotides to hybridize specific lncRNA, and thus magnetic beads coated with streptavidin may pull down oligonucleotide-bound complexes. RNA, DNA and protein would then be isolated and analyzed [16]. Additionally, in order to identify the lncRNA-bound molecular, nucleotide extraction from above strategies could be further assayed by qRT-PCR, qPCR or high-throughput sequencing, and protein can be analyzed by western blot or mass spectrometry. These combinations are named as CHIPR-seq and CHIPR-MS, respectively [15]. To investigate RNA-RNA interactions, cross-linking, ligation and sequencing of hybrids (CLASH) captures structures of dsRNA by using UV cross-linking [17]. Finally, method like RNA-selective 2-hydroxyl acylation and primer extension (SHAPE) is capable of measuring the structure of RNA [18]. Parallel analysis of RNA structure (PARS), together with deep sequencing, simultaneously detect thousands of RNA lengths [19]. In addition, technologies such as RNA analysis on massively parallel array (RNA-MaP), RNA-mechanically induced trapping of molecular interactions (RNA-MITOMI), and cross-linking and immunoprecipitation followed by sequencing (CLIP-seq) are also used to study protein-RNA interactions [15]. Hopefully, flexible combinations of these different assays might continue to extend our knowledge of the RNA world.

**Table 1:** Technologies to study lncRNAs

Purpose	Major approaches
Location	CHIPR; Chart; RAP.
Interactions	CLASH; CHIPR-seq; CHIPR-MS; CLIP-seq; NA-MaPRNA-MITOMI.
Structures	SHAPE; PARS.

## STUDYING lncRNAs IN MOUSE MODEL

The function of numerous lncRNAs have been demonstrated *via* in vitro cell-based assays. Most of them have been further studied using transplanted mouse model rather than genetically engineered mice(GEM). There are couple of reasons for this. Firstly, it is reported that some lncRNA-related GEMs result in embryonic lethality or growth defect. For instance, Sauvageau and colleagues [20] selected 18 different lncRNAs as the targets for genetic deletion in mice and found that 5 knockout lines suffered from destructive outcomes such as death or growth defects. Their results may indicated some lncRNAs may play critical roles in development. Secondly, a few genes editing mice turn into normally developed models, but they later show a inconsistent phenotype with cellular assays. For example, by interbreeding SRA-transgenic mice with the mouse mammary tumor virus-ras (MMTV-ras) transgenic mice, the bitransgenic mice was created which should have produced tumor at a high rate. Instead, the bitransgenic mice later showed the delayed tumor onset compared with MMTV-ras transgenic mice. It is still unclear why SRA seems to disturb ras-induced tumor formation. Although their results indicated that overexpression of SRA promoted cellular proliferation and apoptosis, but insufficient to turmorigenesis [21]. In another case, MALAT1 is believed to regulate process of cancer cell migration, cell cycle progression and alternative splicing, etc. [22]. However, several groups [22–24] have established MALAT1 knockout (KO) model which were surprisingly viable and fertile. MALAT1-depleted cells even failed to show significant changes in nuclear speckle structure and component distribution, and pre-mRNA splicing [25]. These discrepancies between cellular assay and mouse model may be due to reasons as follows. To begin with, lncRNAs sometimes cis-regulate its neighboring genes, or even function like a enhancer, hence depletion of lncRNAs might disrupt nearby genes expression and break local genomic integrity. Moreover, functional redundancy and compensatory mechanisms should be considered in KO model with negative phenotype. Alternatively, the positive phenotypes are more inclined to present in particular condition rather than in laboratory environment with free survival stress. Furthermore, a lncRNA could involve in different mechanisms in different cells. It is necessary to choose the right research approach according to individual condition. Lastly, considering the features of lncRNAs, it is not enough to study them by current adjustment on conventional strategies like whole transcript deletion, exon replacement or insertion leading to a frame shift, etc. [25]. RNA-specific gene editing strategies are urgently needed. More comprehensive introduction of these challenge has been reviewed in [25, 26]. Generally, the study of lncRNAs in mouse model, especially breast cancer-related model, is still infant, future study should develop strategies with higher precision and efficiency.

## lncRNA AND BREAST CANCER

Increasing lncRNAs have been identified to participate in not only physiological processes, but also various cancerous processes, including tumorigenesis, proliferation, apoptosis, invasion and so on [12]. Some lncRNAs even present particular relationship with specific cancer type. For example, overexpression of SCHLAP1 has been associated with metastatic potential and poor prognosis of prostate cancer, and HULC servers as a diagnostic biomarker for hepatocellular cancer [12]. Breast cancer is the top killer in women health. About 249260 new cases and 40890 deaths are estimated in the United States in 2016 [27]. Although great progress has been achieved in understanding the molecular mechanisms of breast cancer, even personalized treatment is developed according to four molecular types (Luminal A, Luminal B, Her2 positive and triple-negative breast cancer (TNBC)), we still faile to reduce the high incidence and overall death rate. So far, several lncRNAs have been reported to be closely related with breast cancer [28–31]. Here, we select seven classic lncRNAs and three novel lncRNAs that have appeared in breast cancer studies, and present a review discussing their mechanisms and clinical application (summarized in Table 2).

**Table 2:** Summary of lncRNAs involved in the breast cancer

LncRNA	Genomic location	Archetype	Function	Cancer phenotype	Breast cancer-pathway examples	Molecular subtype specificity	Possible drug	Reference
HOTAIR	12q13.13	Scaffold, guide, signal	Oncogenic	Proliferation; metastasis; angiogenesis	HOTAIR ⊣miR-568 ⊣NAFT5→ metastasis	Inconsistent	Imatinib/lapatinib	33-40
					HOTAIR ⊣HOXD→ miR7⊣SETDB1/STAT3→ EMT			
MALAT1	11q13.1	Decoy, scaffold	Oncogenic	Proliferation; invasion; migration	MALAT1→ HuR⊣CD133 → EMT (less invasive than TNBC)	Hormone positive	ASOs	47-52
BCAR4	16p13.13	Scaffold	Oncogenic	Proliferation; metastasis; drug resistance	BCAR4→ ERBB2/3 signal pathway→ resistance	Inconsistent	LNAs	53-56
					CCL21→ BCAR4→ non-canonical Hedgehog/GLI2→ migration			
H19	11p15.5	Decoy, scaffold	Oncogenic	Proliferation; metastasis; angiogenesis; apoptosis	H19→ miR-675 ⊣? ⊣Slug ⊣E-cadherin→ EMT	Hormone positive	/	59-61, 70-71
					H19→ miR-675 ⊣c-CbI / CbI-b ⊣EGFR→ proliferation/migration			
SRA	5q31.3	Scaffold	Oncogenic	Proliferation; apoptosis	Unliganded PR→ Repressive complex (containing SRA) ⊣PR induced gene	Hormone positive	/	74-75, 82-83
LINP1	10p14	Scaffold	Oncogenic	IR resistance	EGFR→ RAS–MEK–JNK pathway→ LINP1 → repair of DSBs→ IR resistance	TNBC	/	110
LINK-A	1q43	Scaffold	Oncogenic	Glycolysis reprogramming; tumorigenesis	EGF→ LINK-A→ HIF1α→ glycolysis	TNBC		112
GAS5	1q25.1	Decoy, scaffold	Tumor suppressive	Proliferation; apoptosis; metastasis; drug resistance	GAS5 ⊣GR-induced genes	Inconsistent	Dual PI3K/ mTOR inhibitor; HREM	85-87,90-91,93
					GAS5 ⊣miR-21 → PTEN/TDM1/PCDC4→ proliferation			
XIST	Xq13.2	Guide, signal	Tumor suppressive	Proliferation	XIST→ PHLPP1 ⊣AKT phosphorylation→ cell viability	TNBC	/	95-109
NKILA	20q13	Scaffold	Tumor suppressive	Inflammation; apoptosis; metastasis	MiR-103/107 ⊣NKILA ⊣IkB phosphorylation→ NF-kB	/	/	114

## HOTAIR

LncRNA HOTAIR was first identified by analyzing the results of a tiling array in 2007 [32]. It is a 2158 nucleotide lncRNA located in the locus of HOXC on chromosome 12q13.13, and functions to repress the transcription of the HOXD locus on chromosome 2 in trans. In 2010, Gupta et al. [33] demonstrated a higher amount of HOTAIR in primary breast cancer than in adjacent noncancerous tissue. Later, HOTAIR was found to promote cancer metastasis, and could also serve as a powerful predictor [34, 35].

**Figure 1:**
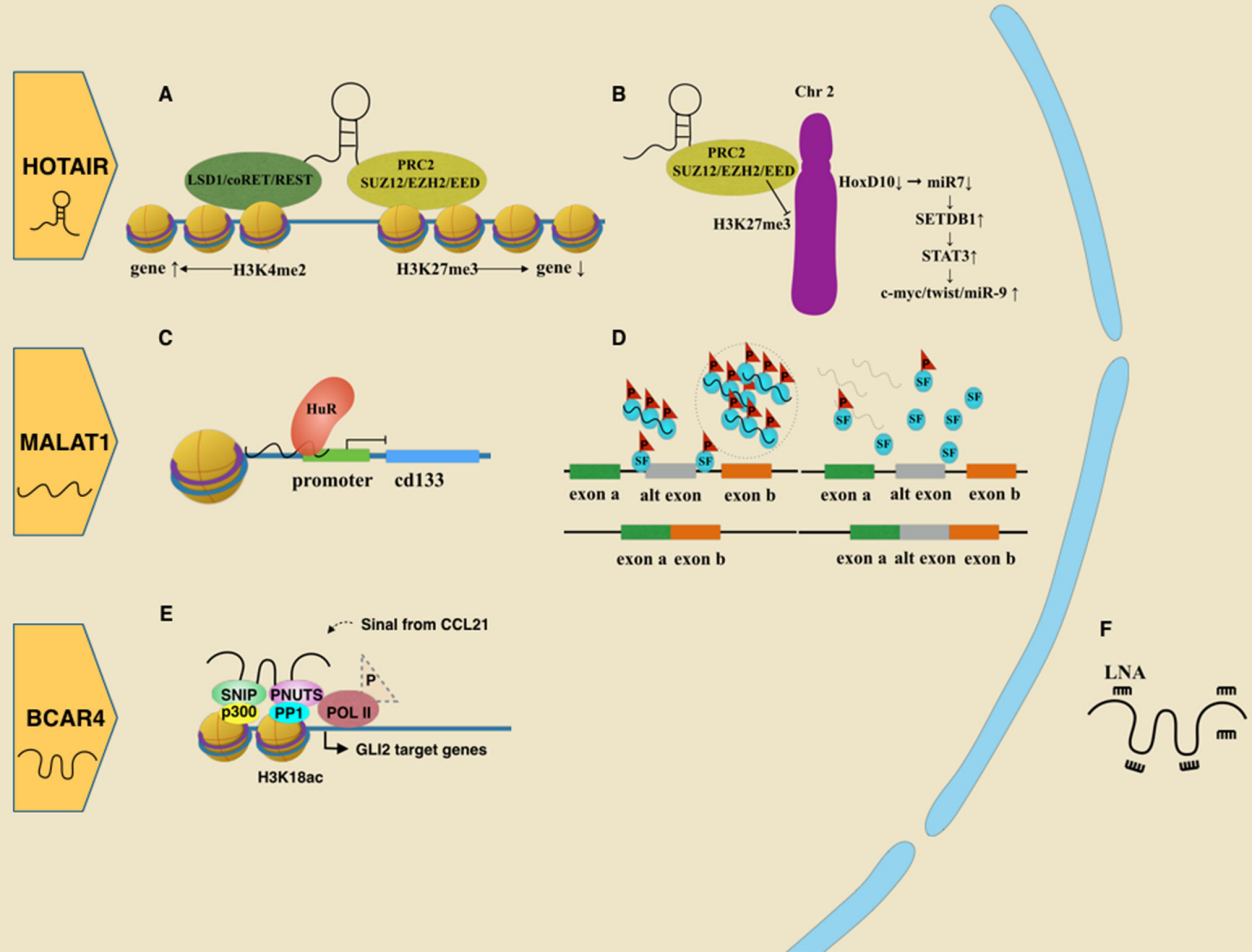

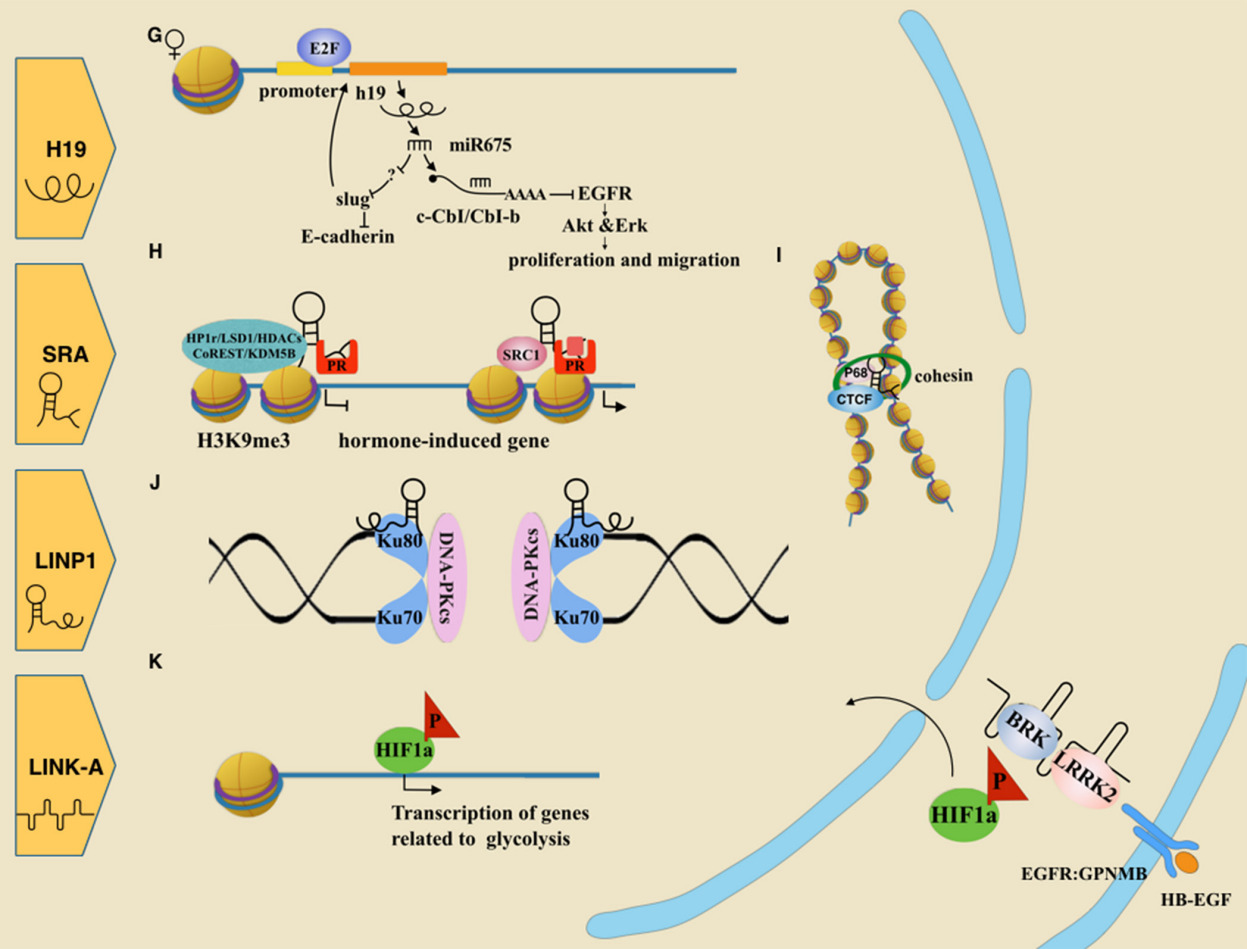
Roles of oncogenic lncRNAs in breast cancer **A**. HOTAIR acts as a scaffold by binding the PRC2 complex or LSD1/coREST/REST complex, leading to gene regulation ***via*** histone modifications. **B**. HOTAIR guides the PRC2 complex to epigenetically repress the HoxD10 locus, which reduces miR7 as well. Reduction of miR7 releases inhibition of SETDB1, which promotes the EMT-related pathway. **C**. MALAT1 negatively controls CD133 transcription *via* HuR. **D**. MALAT1 assembles serine/arginine splicing factors (SF) mainly in the nuclear spectacle. Losing MALAT1 leads SR proteins to be abnormally distributed and abnormally phosphorylated, interfering with proper mRNA processing. **E**. BCAR4 binds with SNIP1 and PNUTS, inducing gene transcription associated with migration. **F**. BCAR4 could be suppressed by LNA. **G**. H19 is the origin of miR675. MiR675 could positively feedback to increase H19 expression by the SLUG/E-cadherin pathway. Alternatively, miR675 protects EGFR by degrading two ubiquitin ligase E3s, facilitating cell proliferation and metastasis through the AKT/ERK pathway. **H**. SRA, as a scaffold, links different complexes to progestin-induced genes when the progestin receptor is not liganded by progestin. **I**. SRA mediates chromosome organization cooperating with CTCF, P68 and the cohesion complex. **J**. LINP1 binds Ku80 and DNA-PKcs as a scaffold, contributing to their function in repairing of DNA double strand breaks. This process also includes molecular Ku70. **K**. LINK-A interacted with both BRK and LRRK2. This complex transfers signal from EGFR:GPNMB heterodimer to nuclear by phosphorylating HIF1α.

Studies addressing the mechanism of HOTAIR show it works as a scaffold to bind functional complexes with several different domains. The 5′ end of HOTAIR interacts with polycomb repressive complex 2 (PRC2), which contains subunits such as SUZ12 and EZH2, and facilitates H3K27 methylation (Figure 1A). Through the interaction, HOTAIR silences targeted genes. In addition, the 3′ end of HOTAIR interacts with the LSD1/CoREST/REST complex, activating genes expression by executing H3K4 demethylation [36] (Figure 1A). Thus, HOTAIR affects multiple downstream genes by modifying specific histone code [32]. For instance, Gupta et al. [33] found elevated expression of HOTAIR led to a gain of PRC2 occupancy and H3K27me3 on 854 genes, which consequently showed changes in gene expression. The Gene Ontology analysis revealed that most of these genes were involved in the pathway of cell-cell signaling and development.

MiR-568 is one of the HOTAIR downstream targets. After being epigenetically silenced, its negative control for nuclear factor of activated T cells 5 (NAFT5) is reduced. NAFT5 is a transcription factor that activates the expression of several metastatic-related genes, such as S100A4 and VEGF-C. Therefore, elevated HOTAIR promotes the epithelial-mesenchymal transition (EMT) and angiogenesis of breast epithelial cells [37]. Another study found that HOXD enhances miR-7 by binding to its promoter. Then, miR-7, which has a complementary sequence to the 3′ UTR of SETDB1 mRNA, might silence SETDB1 and the STAT3 pathway. However, HOTAIR turns over these processes by inhibiting HOXD [38] (Figure 1B).

The relationship between the lncRNA HOTAIR and specifically clinical subtype of breast cancer is still inconsistent. Chisholm et al. [34] identified that HOTAIR correlated with ER and PR positive subtype, which indicated an increasing proliferation rate and a worse outcome. And Xue et al. [39] further confirmed that HOTAIR activates ER without hormone induction, implying a potential role in tamoxifen resistance. However, different from their results, Wang’s group [40] found HOTAIR was most enriched in TNBC tumors. Dual treatments of imatinib and lapatinib, could indirectly down-regulate β-catenin by blocking c-ABL and EGFR. As β-catenin binds at the promoter of HOTAIR, it subsequently represses HOTAIR and suppresses TNBC cell growth. Their discrepancy may due to different research strategies. For example, although Wang’s group extracted RNA from formalin fixed paraffin embedded tissue, their sample size (11 TNBC and 10 non-TNBC) was insufficient. Chisholm’s group showed HOTAIR *in situ* hybridization with synthetic RNA probes. They indeed collected larger amount of samples by making breast carcinoma tissue microarrays. However, most lncRNAs express in low level in specific tissue and RNA degradation may occurr in paraffin-bedded tissue, thus the probe-combination are easily mis-estimated. Some probe-combination might be prevented from stereochemical structure of RNA. To explore this issue, future studies analyzing in large number of fresh tissues are still needed.

## MALAT1

MALAT1, also named nuclear enriched autosomal transcript 2 (NEAT2), locates at chromosome 11q13.1 with a length of 8708 bp. The primary transcript could be cleaved into two parts: a mature 6.7 kb transcript with a short poly(A) tail retained in the nucleus, and a mature 61 nt tRNA-like mascRNA, which is subsequently exported to the cytoplasm with unknown function [41, 42]. MALAT1 was originally identified and proved to be correlated with metastasis in non-small cell lung cancer patients [43]. Later, accumulated evidence has shown that MALAT1 is elevated in a broad spectrum of cancer types, including breast cancer [44, 45].

In cancer cell, nuclear MALAT1 is involved in proliferation, invasion and migration. A classic function is to regulate the alternative splicing of pre-mRNA by interacting with serine/arginine (SR) splicing factors (SF) [46]. In normal cells, many SR proteins stay in nuclear speckles in hyperphosphorylated form. However, in cells with MALAT1 depletion, there would be an increase in both cellular SR proteins level and ratio of dephosphorylated SR proteins to phosphorylated SR [46] (Figure 1D). Additionally, MALAT1 was found to form a repressive complex with the RNA-binding protein HuR, which negatively controls CD133 gene expression by binding to its promoter (Figure 1C). CD133 is a marker for cancer stem cells and promote the EMT-program in various cancers. Its superior level in TNBC than in ER positive breast subtype, indicates that MALAT1 is more highly expressed in ER positive cell lines [47]. This ER-related expression has been supported both in full-length MALAT1 and an alternatively spliced variant of MALAT1 [48, 49]. By interacting with polycomb 2 protein, MALAT1 could mediate gene activation by relocating growth-related genes as well [50]. Recently, an exciting study showing therapeutic potential of MALAT1 on mouse model is getting attention. By crossbreeding MALAT1 KO female mice with male mice with high incidence of mammary tumor (MMTV-PyMT mice), three filial generations were obtained (MMTV-PyMT Malat1+/+; MMTV-PyMT Malat1+/−; MMTV-PyMT Malat1−/− mice). In MALAT1-deficient filial mice, tumors show cystic form with better differentiation and less aggressiveness, while in filial mice with normal levels of MALAT1 expression, tumors tend to be solid carcinomas with more poorly differentiation and more aggressiveness. Then, the authors injected anti-Malat1 antisense oligonucleotides (ASOs) into above filial mice, which surprisingly turned the tumors in MALAT1 positive mice into cystic form, similar to those in MALAT1-deficient type [51]. Their findings provide us powerful confidence in exploration of lncRNA [52].

## BCAR4

Infecting estrogen-dependent human ZR-75-1 cell line with retroviral transduction of cDNA libraries, which derived from human placenta/brain or mouse embryo, will cause tamoxifen-sensitive cell to develop a tamoxifen-resistant phenotype. Among those different genes, breast cancer anti-estrogen resistance 4 (BCAR4) was found to be closely related to tamoxifen resistance [53]. Godinho et al. [54] later confirmed BCAR4-mediated resistance and found its independence of estrogen receptor 1 (ESR1) expression. In contrast, overexpression of BCAR4 led to strong phosphorylation of ERBB2 and ERBB3, indicating an involvement of ERBB2 signaling pathway. Moreover, by performing immunoprecipitation, they identified that the ERBB2/3 signal pathway drove the BCAR4-mediated tamoxifen resistance [55].

Besides, BCAR4 also causes anchorage-independent cell growth, which promotes tumor metastasis and poor overall survival [53]. It contributes to tumor migration by mediating transcription of glioma-associated oncogene homolog 2(GLI2)-dependent target gene in a noncanonical Hedgehog-GLI pathway. Extracellular chemokine CCL21 transmits its signal into cell, indirectly recruiting BCAR4 to bind with Smad nuclear-interacting protein 1 (SNIP1). Since SNIP1 is an inhibitor for p300, its binding with BCAR4 would liberate histone acetyltransferase (HAT) activity of p300, leading to acetylation of histones such as H3K18ac. Moreover, BCAR4 could also recruit Serine/threonine-protein phosphatase 1 regulatory subunit 10 (PPP1R10, also known as PNUTS). PUNTS, which originally functions to inhibit the phosphatase activity of PP1, is negatively affected by H3K18ac and subsequently in turn attenuates the inhibitory effect on PP1. The accumulated PP1 dephosphorylates RNA Pol II Ser5, resulting in transcription of GLI2 target genes [56] (Figure 1E). Due to the important role of BCAR4 in CCL21-induced hypophosphorylation of RNA Pol II Ser5, locked nuclear acid (LNA) has been used to target BCAR4 (Figure 1F) and suppress metastasis of breast cancer in mouse models [56].

## H19 AND 91H

H19 is an imprinting lncRNA that is exclusively transcribed from the maternal allele in humans [57, 58]. It locates on chromosome 11p15.5 and shares enhancer with a neighboring gene igf2, which is a reciprocally paternal imprinting gene. As an oncogene, its overexpression facilitates MDA-MB-231 cells to form more and larger colonies in soft-agar than the control cells. And nude mice injected with H19-transfected cells, develop increasing tumor progression [59, 60]. A novel technology, chromogenic *in situ* hybridisation (CISH), has been used to identify and localize lncRNAs [34, 61]. It shows a significantly overexpression of H19 in either invasive breast cancer (IBC) or ductal carcinoma *in situ* (DCIS) compared with normal adjacent breast tissues (*p* < 0.05) [61].

H19 is involved in multiple stages of tumor progression including proliferation and metastasis [62]. During tumor proliferation, it down-regulates suppressors such as p57kip2 or up-regulates oncogenes such as cyclin E2. As a result, H19 facilitates transcription of angiogenic genes or inhibits apoptotic-related genes. In addition, transcription factor E2F elevates H19 by binding its promoter, leading to accelerated G1-S transition and cell cycle progression [63]. Additionally, H19 is also involved in the tumor metastasis, which includes two converse events: the EMT and the MET. When the epithelial cells migrate through the extra cellular matrix *via* EMT, blood flow carries them to a susceptible site. Then, the tumor cells develop into a secondary lesion by the converse process of MET [64]. In the regulation of EMT, Matouk et al. [65] identified a link among H19/miR-675, Slug and E-cadherin (Figure 1G). MiR-675 is a conservative transcript derived from lncRNA H19 [66]. It could suppress c-CbI and CbI-b, two ubiquitin ligase E3s that function to degrade EGFR (Figure 1G). Consequently, accumulated EGFR activates cell proliferation and migration through the Akt and Erk pathway [67]. Another transcript miR-200 also participates in MET process in malignant breast cells [68]. Its close relationship with H19 has been observed in hepatocellular carcinoma [69]. Does their “sponge”-like behavior also work in breast cancer? Further research is needed to answer it. Together, though H19 is involved in both EMT and MET, its final phenotype might depend on the cellular context and the specific EMT-MET balance [62].

An estrogen-ERa-H19 signaling axis is demonstrated in ER positive breast cancer [70]. LncRNA H19 in ER-positive breast cancer shows 10 times more than that in ER-negative tumor tissues [71]. The elevated expression could be induced by 17beta-estradiol *via* the ERa pathway, eventually promoting survival and proliferation of MCF-7 cells [71]. Consistently, another study observes that blocking ERa in luminal progenitors led to a decline of H19 expression and smaller colony formation, which is similar to the result of H19 knocking down [70]. This estrogen-ERa-H19 signaling axis might inspire treatment options for ER positive breast cancer.

Within the H19/igf2 loci, there is another lncRNA 91H, which is antisense to the H19 gene. The 91H is also up-regulated in human breast tumors, and knockdown experiments show that 91H affects igf2 expression in trans [72].

## SRA

The steroid receptor RNA activator (SRA) was initially found to be a non-coding RNA. It selectively modulates the function of steroid receptors, such as ER [73] and PR [74]. Specifically, it mediates transactivation by binding with the N-terminal AF1 (activation function 1 domain) of steroid receptors and forms a co-active complex with SRC-1 (steroid receptor co-activator 1) [74]. SRA also participates in the repressor complex and silences those hormone-induced genes prior to ligand binding. For instance, SRA acts as a scaffold to form a repressive complex with its partners, including HP1r, LSD1, HDAC1/2, CoREST, and KDM5B. This complex is anchored to hormone-induced genes when the progesterone receptor is unliganded in breast canner, but displaced after hormone treatment [75] (Figure 1H). Analysis of bioinformation shows that most SRA-affected genes are associated with cell proliferation and apoptosis [21]. In addition, SRA, together with the RNA helicase P68, participates in CTCF-mediated chromosome organization [76] (Figure 1I). It is also a part of nuclear receptor-mediated transcription and miRNA processing [77].

There are several mechanisms modulating the function of SRA. One of them is that pseudouridine synthase family members, Pus1p and Pus3p, transform uridine into pseudouridine, thus altering the structure of SRA and controlling its co-active or co-repressive role [78]. Moreover, SRA can be inhibited by SLIRP and SHARP [79, 80].

The level of SRA in normal breast tissue is much lower than that in tumor tissue [74]. Several SNPs (rs10463297, rs801460) located in whole SRA sequence has been identified to increase cancerous risk [81]. SRA not only acts as a regulatory lncRNA, but also codes for the SRA protein (SRAP), which is highly expressed in primary breast tumors as well [82, 83]. The balance between fully-spliced SRAP-coding RNA and intron-1-containing non-coding SRA RNA might characterize the specific phenotypes of breast cancer and modulate tumorigenesis and progression by regulating the expression of specific genes [84].

## GAS5

Unlike above lncRNAs, GAS5 is considered to be an oncological suppressor. Its coding gene GAS5, which locates at chromosome 1q25.1, is one of the 5′ terminal oligo-pyrimidine class genes. Low expression of GAS5 was identified in multiple cancers, including breast cancer, prostate cancer, colorectal cancer and lung cancer [85]. It involves in cell proliferation, apoptosis process and trastuzumab resistance [86, 87].

**Figure 2:**
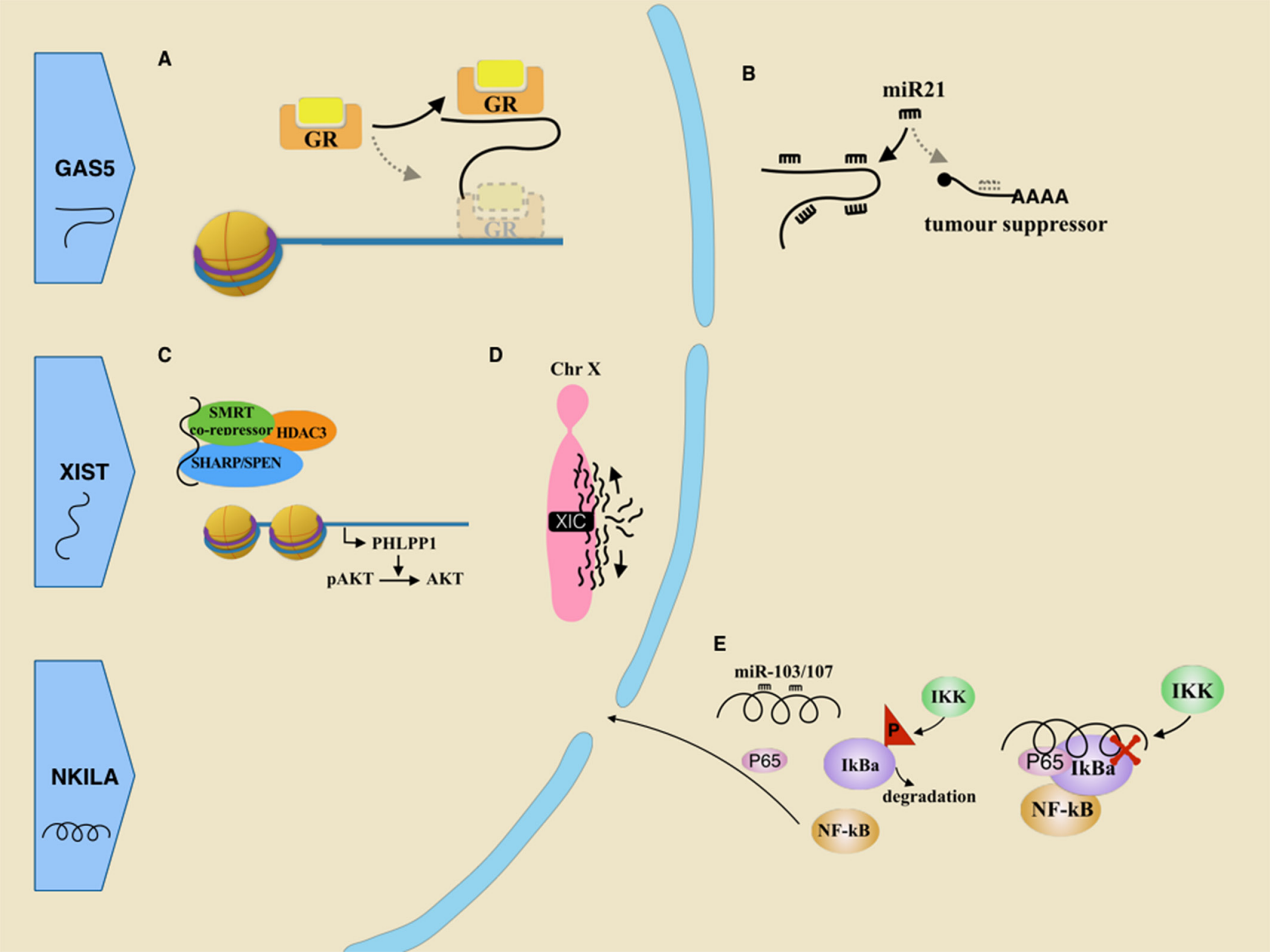
Roles of tumor suppressive lncRNAs in breast cancer **A**. GR mistakenly recognizes GAS5 as its DNA target, leading to GR sink. **B**. As a molecular sponge, GAS5 inhibits miR21 mediated mRNA degradation of some tumor suppressors. **C**. XIST indirectly recruits HDAC3, stopping its disturbing PHLPP1 transcription. PHLPP1 functions to dephosphorylate pAKT. **D**. XIST, derived from the X inactivation center (XIC) at one of two X chromosomes, spreads along the X chromosome and eventually silences it. In cancer-related inflammation, lncRNA NKILA could be repressed by miR-103/107 so that it fails to cover the phosphorylation site of IkB. Thus IkBa is phosphorylated and degraded, which activate NF-kB pathway.

Owning a 5′ terminal oligopyrimidine (5′ TOP), GAS5 mRNA is stimulated by mTOR, which promotes its own translation [88]. After translation, the GAS5 mRNA is degraded through the nonsense-mediated RNA decay (NMD) pathway [89]. This whole process is active in normally growing cells, which results in a lack of surplus GAS5. However, in growth arrested cells, the decrease of mTOR leads to GAS5 accumulation [88]. Surplus GAS5, which mimics the structure of the glucocorticoid response element (GRE), competes with the real DNA GREs for binding to the glucocorticoid receptor (GR) (Figure 2A). Overall, it silences GR and suppresses the glucocorticoid-induced genes, including one that encodes for an inhibitor of apoptosis 2.

Reduction of GAS5 will attenuate drug-induced death in breast cancer cells. Modulating apoptosis by GAS5 has been recognized as a potential explanation for resistance to chemotherapy [90]. To solve this problem, Pickard et al. suggested either to use drugs that bypass the GAS5 pathway or to increase GAS5 by dual PI3K/mTOR inhibition [90]. Moreover, the Pickard’s group induced apoptosis in breast cancer cells by giving exogenous hormone response element mimic (HREM) [91]. It simulates a stem-loop structure in the 3′ termination sequence of lncRNA GAS5, which is required for decoy-binding [92]. Another GAS5 mechanism as a suppressor is in light of its reciprocal inhibition on miR-21 [87, 93]. MiR-21 is overexpressed in many tumor tissues and is considered to regulate cancerous processes, such as cancer development and metastasis. First, GAS5 might act as a sponge to interact with miR-21 because of a specific binding site in its exon 4. Silencing miR21 suppresses the transcription of several downstream genes, including PTEN, TDM1 and PCDC4 (Figure 2B). Second, it is also known that miR-21 might down-regulate GAS5 expression *via* RISC in breast tumor specimens [93] (Figure 2B).

## XIST

XIST is a 19 kb non-coding transcript that is expressed from an early developmental stage. It is mainly transcribed from the X chromosome destined to be Xi (the inactive X chromosome), and it spreads along this X chromosome (Figure 2D). The process of X chromosome inactivation (XCI) occurs in early embryogenesis, which includes counting, choosing X chromosome homologs and the initiation, propagation, and maintenance of Xi [94]. Approximately one thousand genes are thus silenced, leading to dosage compensation between the two genders. However, loss of X inactivation or XIST transcript has been noticed in breast and ovarian cancer [95].

In breast cancer, the lncRNA XIST was significantly reduced comparing with normal breast tissue [96]. With the help from SHARP/SPEN and SMRT co-repressor, XIST functions as a decoy to indirectly titrate histone deacetylase 3 (HDAC3) from the promoter of PH domain and leucine-rich repeat phosphatase 1 (PHLPP1), so that enough PHLPP1 is transcribed. PHLPP1 is a phosphatase which turns active pAKT into AKT, resulting in limited cell viability. However, reduction of XIST level in breast cancer releases HDAC3 which can target and suppress the promoter activity of the PHLPP1 [96] (Figure 2C).

It has been found that RNAs might down-regulate the expression of XIST expression. For instance, Tsix RNA represses XIST transcription by facilitating a germline factor, PRDM14, to bind to intron 1 of XIST [97]. Additionally, proteins such as BRCA1, NANNOG and OCT4 also involve in the regulation [98]. BRCA1 was initially found to co-localize with an inactive X chromosome in breast cancer cells. Subsequently, inhibition assays supported that loss of BRCA1 in female cells might lead to Xi perturbation and the dysregulation of lncRNA XIST [99]. BRCA1 is a tumor suppressor helping to repair damaged DNA. Loss of its function leads to a strong susceptibility for breast cancer. Later, Ganesan’s et al. further demonstrated that BRCA1 functioned to maintain proper Xi heterochromatin [100]. Another study also reported that the loss of XIST RNA was associated with BRCA1 deficiency in sporadic basal-like cancers (BLC) [101]. However, in contrast to these researches, several labs failed to find a location relationship and interaction between BRCA1 and XIST RNA [102–104]. The contradiction may be due to multiple Xi absent possibility. There exists at least three different patterns (loss of Xi without gain of Xa; loss of Xi with sequent reactivation; and loss of Xi accompanied with replication of Xa) and XIST RNA can also be transcribed from Xa and Xi [105, 106], hence further studies are needed to elucidate this complicated issue. Some investigators found that the frequent deficiency of X chromosome inactivation (XCI) might be independent of XIST expression and BRCA1 status [105].

Recently, XIST lncRNA was identified as a biomarker for predicting the drug response to the histone deacetylase inhibitor abexinostat. By evaluating the level of XIST lncRNA, breast cancer cell lines could be divided into a low-dose sensitive group whose cancer stem cells (CSC) were differentiated by abexinostat, and a high-dose sensitive group whose CSC population were stable [107]. Moreover, expression of XIST also contributed to predict the sensitivity to chemotherapy, such as the high-dose alkylating agent cisplatin [108, 109].

## SOME NOVEL lncRNAs

The study exploring novel lncRNAs is progressing rapidly. Here, we select three of them to elaborate. LncRNA LINP1 is associated with the non homologous end joining pathway (NHEJ) in TNBC (Figure 1J) by acting as a scaffold and linking Ku80/70 and DNA-PKcs to broken ends [110]. Due to the role of NHEJ in radiotherapy resistance [111], it is interesting to inquire the relationship between LINP1 and radiotherapy. Results show that the repression of LINP1 would sensitize tumors to radiotherapy [110]. Furthermore, in the glycolysis reprogramming of TNBC, long intergenic non-coding RNA for kinase activation (LINK-A) also plays an important role [112]. Receiving signal triggered by heparin-binding EGF (HB-EGF), LINK-A interacts with breast tumor kinase (BRK) and leucine-rich repeat kinase 2 (LRRK2), facilitating their phosphorylation on HIF1α. Active HIF1α then starts transcriptional programs and promotes glycolysis reprogramming in TNBC [112] (Figure 1K).

Inflammation in tumor microenvironments has been identified to promote tumor invasion and metastasis. Nuclear factor-kB (NF-kB) plays a crucial role in inflammation [113]. This family of transcription factors mediates transcription of many chemokines and cytokines. Additionally, it also transcribes many negative regulators for the NF-kB pathway, and one of its targets is lncRNA NKILA [114]. In normal cells, lncRNA NKILA could mask the phosphorylation site in inhibitor of NF-kB (IkB) with the help of P65, preventing its phosphorylation by IkB kinases (IKK), thus negatively regulating NF-kB activation. But in tumor cells, NKILA would be a target for miR-103/107 and indeed silenced by miRNA-mediated RNA degradation (Figure 2E). Not only has it been found to attenuate inflammation, but it is also known to suppress metastasis in breast cancer. And low expression level of NKILA usually predicts poor prognosis [114].

## CLINICAL APPLICATIONS

With a deepening understanding of lncRNAs, their potential to be biomarkers is attracting increasing attention [115–117]. Specifically, lncRNAs not only act as diagnostic or prognostic markers but also are the targets for treatment. To discover diagnostic markers, MingmingLv et al. [118] detected the dysregulated lncRNAs in TNBC and non-TNBC sample through microarray technique. Gene Ontology and Pathway analysis indicated the possible function of differentially expressed lncRNAs. Finally, they validated 4 potentially diagnostic lncRNAs (RP11-434D9.1, LINC00052, BC016831, and IGKV) through receiver operating characteristic curve analysis. As a prognostic marker, BCAR4 could predict tamoxifen resistance and tumor invasion [119]. Meanwhile, Gupta et al. [33]found nearly one third of breast tumors expressed elevated HOTAIR compared to normal breast epithelia. This high level HOTAIR predicted a tendency for metastasis and death (*p* < 0.01). Furthermore, HOTAIR has also been validated as a good radio-genomic biomarker. Increasing HOTAIR is associated with a higher ERF (enhancing rim fraction) score, a feature found by dynamic contrast material-enhanced (DCE) breast magnetic resonance (MR) imaging. Elevated ERF scores indicate an earlier metastasis of breast cancer and a worse metastasis-free survival. The tendency between HOTAIR and ERF might occur because of the mechanism that HOTAIR promotes metastasis and vascular growth [120]. Not only can individual lncRNA serve as a single predictor but also a group of lncRNA shed light on our understanding of breast cancer. Liu et al. [121, 122] proposed an mRNA-lncRNA signature to independently predict TNBC. They selected RNAs that significantly correlated with patients recurrence-free survival (RFS) and constructed signatures relying on their coefficients in the multivariate Cox proportional hazards regression model. This integrated mRNA-lncRNA signature could calculate recurrence risk score according to the formula as follows: 0.939*ABCA8- 2.593*CHRDL1+0.517*ADH1B-0.329*CDK1-0.071*CDC6+0.02*SQLE-1.146*FCGR1A+1.366*RSAD2+0.361*SNRPEP4+0.277*HIST2H2BC. The former eight RNAs are mRNAs and the latter two are lncRNAs. Comparing to another mRNA-only signature ( Recurrence risk score = 0.877*ABCA8-2.553*CHRDL1+0.531*ADH1B-0.238*CDK1+0.086*CDC6+ 0.219*SQLE-1.14*FCGR1+1.380RSAD2), the integrated mRNA-lncRNA signature performs better in predicting recurrence risk and the benefit of taxane chemotherapy in patients with TNBC. Recently, they have applied for a clinical trial (NCT02641847) validating whether this mRNA-lncRNA signature is a good biomarker for predicting risk of recurrence and choosing proper treatments for subsequent chemotherapy (https://clinicaltrials.gov). The study is now recruiting eligible invasive TNBC patients. By testing their samples using RT-PCR (real-time polymerase chain reaction) and recording individual recurrence risks, the patients will be divided into a high risk group and a low risk group accepting different treatments. Furthermore, there are several clinical trials trying to verify the role of lncRNA in other diseases (NCT02602808, NCT02304471), such as acute pancreatitis, CKD, and ESRD. All of these results and future studies will bring significant development in the future of lncRNAs.

LncRNAs can not only act as predictive biomarkers but also be promising targets for treatment. ASOs, which are complementary to certain lncRNA, might decrease lncRNA function. As we mentioned above, Arun et al. [51] used ASO to down-regulate MALAT1 in MMTV-PyMT mice or their tumor organoids, and observed a reduction of tumor proliferation and metastasis. Moreover, LNA-based ASOs targeting BCAR4 might serve as a treatment strategy as well [56]. Recently, some lncRNAs have been found to encode a micropeptide when translated from a short ORF [8]. For instance, myoregulin, encoded by skeletal muscle related non-coding RNA, shares a similar domain with some Ca2+ regulators. It can regulate the Ca2+ concentration in the sarcoplasmic reticulum [123]. If the functional micropeptide coded from lncRNA proves ubiquitous, this may open a new area for studying cancer.

Overall, current knowledge about lncRNAs is still infant. Although the lncRNAs we discussed above participate in various processes of breast cancer, few of them are breast-unique. Further studies are needed to investigate lncRNAs with more specificity.

## CONCLUSIONS

In conclusion, lncRNAs play a vital role in breast cancer study for the following reasons. 1) As transcripts, lncRNAs are generally synthesized faster than proteins. 2) LncRNAs can interact with both nucleotide sequences and proteins. 3) Because they are larger than 200 nucleotides, lncRNAs can form more complex structures than microRNAs, thus carrying out more complicated functions. 4) LncRNAs have been demonstrated to affect various processes in breast cancer events, such as proliferation, metastasis, angiogenesis, drug resistance, etc. With more RNA-centric approaches being developed, a better understanding of lncRNAs will help us in preventing, diagnosing, and treating breast cancer.
